# Targeting CD38 in Antibody-Mediated Rejection

**DOI:** 10.3389/ti.2025.14343

**Published:** 2025-05-15

**Authors:** Katharina A. Mayer, Klemens Budde, Matthias Diebold, Philip F. Halloran, Georg A. Böhmig

**Affiliations:** ^1^ Division of Nephrology and Dialysis, Department of Medicine III, Medical University of Vienna, Vienna, Austria; ^2^ Department of Nephrology, Charité Universitätsmedizin Berlin, Berlin, Germany; ^3^ Clinic for Transplantation Immunology and Nephrology, University Hospital Basel, University of Basel, Basel, Switzerland; ^4^ Alberta Transplant Applied Genomics Centre, Faculty of Medicine and Dentistry, University of Alberta, Edmonton, AB, Canada

**Keywords:** antibody-mediated rejection, CD38, natural killer cells, kidney transplantation, treatment

## Abstract

Antibody-mediated rejection (AMR) remains a major challenge in clinical transplantation. Current therapies have yielded inconsistent outcomes, highlighting the need for innovative approaches. CD38, a multifunctional glycoprotein, is highly expressed on plasma cells and natural killer (NK) cells, potentially offering a dual mechanism of action that could intervene in the pathophysiologic course of AMR: depleting alloantibody-producing plasma cells and NK cells. This review focuses on recent results from CD38-targeted therapies, with felzartamab emerging as a promising option. Previous case reports and series suggested that off-label daratumumab treatment could effectively reverse AMR. Felzartamab has now demonstrated safety and efficacy in a phase 2 trial for late AMR. Reductions in microvascular inflammation, downregulation of rejection-associated transcripts, and decreases in donor-derived cell-free DNA paralleled a substantial decrease in NK cell counts. However, felzartamab did not significantly affect donor-specific antibodies, which may reflect its distinct mechanism of action, primarily involving antibody-dependent cellular cytotoxicity and phagocytosis. The effects on rejection activity may have a rapid onset, but are transient. The potential benefits of prolonged therapy are currently being investigated in a recently launched phase III trial. Future studies may expand the applications of CD38 targeting to early AMR or broader indications, such as DSA-negative microvascular inflammation.

## Introduction

Antibody-mediated rejection (AMR) after kidney transplantation is a major clinical challenge [1, 2], often leading to chronic injury and declining graft function, which contributes to poor graft survival [3–5]. Despite advances in immunosuppression, AMR remains a frequent cause of allograft failure [6, 7], and there are currently no approved therapies, highlighting the urgent need for effective treatments [8, 9].

During the pathophysiologic course of AMR, donor-specific antibodies (DSA) arise from alloantigen-specific T cell-dependent B cell activation. This process generates a reservoir of donor-specific memory B cells and/or plasma cells [10, 11]. DSA produced by plasma cells may bind to HLA molecules on the surface of the allograft endothelium, inducing microvascular inflammation (MVI), the histologic hallmark lesion of AMR [12]. Beyond complement activation, DSA can also mediate graft damage through direct signaling and Fc-mediated effector mechanisms, including the binding of the Fc portion of DSA to Fc receptors (FcR) on innate immune cells, such as CD16^+^ natural killer (NK) cells and monocytes/macrophages [10, 11, 13, 14].

Over recent decades, research into novel therapies for AMR has focused on identifying targets to reduce DSA levels and impair plasma cell function. However, controlled studies investigating treatments that target various aspects of B cell and plasma cell immunity have failed to demonstrate clear therapeutic benefits [8, 9]. This is particularly evident for late (active and chronic active) AMR, where interventions aimed at plasma cell generation and/or integrity (e.g., interleukin-6 blockade [15] or proteasome inhibition [16]), DSA removal (e.g., immunoglobulin G degradation using imlifidase [17]), complement inhibition [18], or depletion of early B cell populations (e.g., rituximab in combination with intravenous immunoglobulin [19]) have not produced convincing outcomes. According to the Transplantation Society Working Group, optimizing baseline immunosuppression is the primary recommendation for late AMR, while apheresis combined with intravenous immunoglobulin remains the standard for early active AMR, despite limited evidence [8].

Recently, novel treatment strategies have emerged, with the most promising being the targeting of CD38 via monoclonal antibodies, as supported by the positive results from a recent phase 2 trial of the CD38 antibody felzartamab [20, 21]. The application of CD38 antibodies has broadened therapeutic options beyond plasma cell-directed therapies to a multifaceted strategy that includes simultaneous depletion of plasma cells and innate effector cells [20–24].

In this review, we summarize the current evidence supporting monoclonal CD38 antibodies as a novel therapeutic option for AMR and discuss their potential future applications, including HLA desensitization.

## CD38 – A Multifunctional Molecule

The complexity of CD38 is detailed in recent reviews, emphasizing its diverse and often poorly understood biological roles, such as in infection defense, chronic inflammation, and autoimmunity [25]. Immunologically, CD38 regulates cell differentiation, proliferation, cytokine release, apoptosis, phagocytosis, chemotaxis, and transmigration, the latter potentially involving selectin-like binding of hematopoietic cells to endothelial cells via CD31 [25]. CD38 is a non-lineage-restricted, single-chain transmembrane glycoprotein comprising 300 amino acids with a molecular weight of 45 kDa. It lacks an internal signaling domain and is encoded in humans on chromosome 4. First identified in the early 1980s, CD38 was initially described as a surface protein on T cells capable of inducing cell activation [26]. CD38 is constitutively expressed and upregulated upon activation in various immune and hematopoietic cells (T cells, B cells, NK cells, dendritic cells, etc.) and precursors. CD38 is also found in tissues like the prostate, pancreas, smooth muscle, kidney, gut, and brain [25].

Over recent years, CD38 has gained attention as a marker and therapeutic target in hematopoietic malignancies, particularly multiple myeloma [27]. In organ transplantation, its high expression on plasma cells and NK cells suggests a dual mechanism for CD38-targeting antibodies in immune cell depletion [28]. However, this view may oversimplify the complex physiology of CD38. Such antibodies might also modulate enzymatic activity or affect the function and activation of other immune cell subsets, including regulatory cells.

Interestingly, CD38 shares striking molecular similarity with an enzyme from the mollusk *Aplysia californica*. This resemblance has led to its identification as a NAD-depleting ectoenzyme with ADP-ribosyl cyclase and hydrolase activities. CD38 converts nicotinamide adenine dinucleotide (NAD^+^) into nicotinamide and cyclic ADP-ribose (cADPR), which is then hydrolyzed to ADP-ribose (ADPR), but its enzymatic function has turned out to extend much farther [29, 30]. Expressed on cell surfaces and in intracellular compartments, CD38 has two orientations: type II and type III, the latter positioning the catalytic domain toward the cytosol and implicating CD38 in intracellular NAD^+^ regulation, vital for mitochondrial function and metabolism. Its enzymatic activity also links CD38 to intracellular signaling, as ADPR and cADPR serve as second messengers that regulate Ca^2+^ levels. This enzymatic versatility highlights potential roles in health and disease, with NAD homeostasis changes contributing to various pathologies [25, 31, 32].

Of particular relevance to AMR, where NK cells have recently garnered interest [10, 11, 14], is the role of CD38 as a receptor that regulates NK cell cytokine release and cytotoxicity [33, 34]. Experiments with interleukin-2-activated NK cells revealed that ligation of CD38 with agonistic monoclonal antibodies significantly increased intracellular Ca^2+^ levels and induced tyrosine phosphorylation of cytoplasmic substrates, resembling activation via FcγRIIIA (CD16) [34]. CD38 engagement also elevated HLA class II and CD25 expression, promoted IFN-γ and granulocyte-macrophage colony-stimulating factor release, and enhanced cytolytic effector functions against target cells [34]. A series of experiments showing surface proximity between CD38 and CD16 suggests that signaling via CD38—despite lacking the canonical receptor structure—is enabled by functional and physical associations with another professional signaling structure, such as CD16 in NK cells [35, 36].

### Monoclonal CD38 Antibodies–Applications in Organ Transplantation and Beyond

Several CD38 antibodies have been developed for the treatment of multiple myeloma, where they have been an established option with an acceptable safety profile for many years [37]. Recently, three CD38 antibodies—felzartamab, daratumumab, and isatuximab—have been tested in organ transplantation, particularly for the treatment of AMR and desensitization in broadly HLA-sensitized recipients.

*Felzartamab* (MOR202; IgG1λ) has shown efficacy in relapsed or refractory multiple myeloma [38], and is now being developed for autoimmune diseases, including membranous nephropathy [39] and IgA nephropathy (ClinicalTrials.gov identifier, NCT05065970), as well as AMR in kidney transplants, with encouraging results in a phase II trial [20, 21].

*Daratumumab* (IgG1κ), the first monoclonal CD38 antibody approved for multiple myeloma treatment [40, 41], has been explored off-label in AMR, with several case reports and series published to date [42–50]. In addition, studies have been conducted in transplant recipient desensitization, and outside transplantation, such as in autoimmune diseases [51–53]. A recently proposed indication in the transplant setting may be FSGS recurrence, as suggested by recent case series in which daratumumab, was successfully used in combination with CD20 antibodies rituximab or obinutuzumab [54, 55].

*Isatuximab* (SAR650984; IgG1κ) was developed for multiple myeloma [56]. It has since been studied in transplant recipient desensitization [57, 58], though no data currently exist on its use in established AMR.

The CD38 antibody CID-103 was recently considered for AMR treatment but placed on clinical hold by the FDA. Mezagitamab, is being investigated for systemic lupus erythematosus [59] and IgA nephropathy (ClinicalTrials.gov identifier, NCT05174221), with a phase 3 trial for ITP underway (NCT06722235). Additionally, CM313 has demonstrated rapid platelet count increases in ITP by inhibiting ADCC on platelets while maintaining long-term efficacy via plasma cell clearance [60]. However, none of these antibodies are currently being evaluated in the organ transplant context.

### Molecular and Cellular Effects of Targeting CD38

**FIGURE 1:**
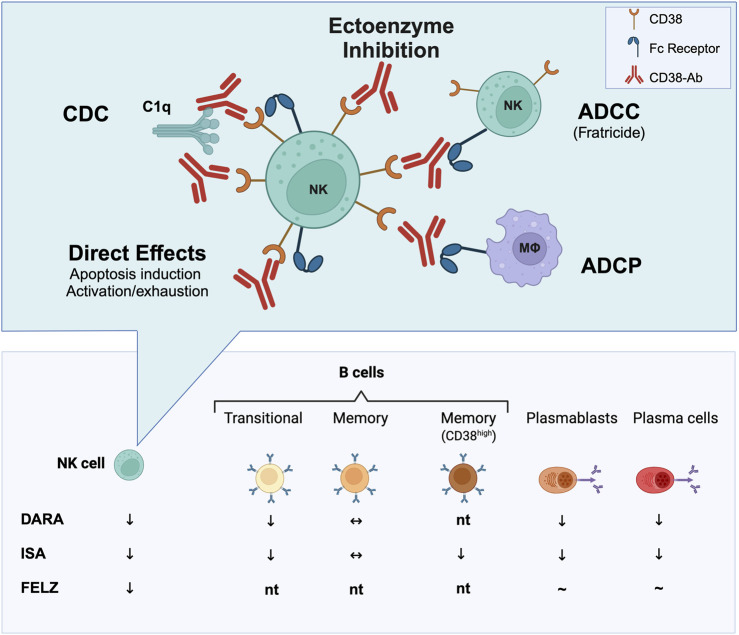
Immune cell depletion through CD38 targeting. In transplant studies (e.g., rejection treatment or recipient desensitization), CD38 antibodies such as daratumumab (DARA), isatuximab (ISA), and felzartamab (FELZ) have been shown to deplete various immune cell (sub)types in peripheral blood, bone marrow and/or transplanted kidneys, most consistently natural killer (NK) cells. Isatuximab, in particular, has demonstrated the ability to deplete CD38^+^ memory B cells, plasmablasts, and plasma cells. Conversely, felzartamab has not been convincingly shown to deplete CD138-positive cells in peripheral blood, though its effects on distinct B cell subsets and plasma cells in the bone marrow have not yet been tested (nt). CD38 antibodies may exert functional effects through multiple mechanisms. For NK cells—key effector cells involved in antibody-mediated rejection—these mechanisms potentially include inhibition of the ectoenzymatic function of CD38 or depletion of target cells through Fc-mediated processes. These processes encompass antibody-dependent cellular cytotoxicity (ADCC), leading to NK cell fratricide; antibody-dependent phagocytosis (ADCP); complement-dependent cytotoxicity (CDC) via the attachment of the key complement component C1q; as well as apoptosis or NK cell exhaustion upon activation. Created with Biorender.com.

Analyses of CD38 antibody mechanisms, primarily from preclinical myeloma models suggest that target cell depletion involves Fc-dependent immune effector processes, including complement-dependent cytotoxicity (CDC), antibody-dependent cellular cytotoxicity (ADCC), and antibody-dependent phagocytosis (ADCP) [27] Additional mechanisms may include interference with CD38’s ectoenzymatic activity, apoptosis induction, or triggering CD38-dependent activation, potentially affecting immune cells like NK cells, where overactivation may cause exhaustion or cell death. Possible modes of action of CD38 antibodies, illustrated using NK cells as target cells, are shown in Figure 1. Different CD38 antibodies use distinct mechanisms. Felzartamab primarily mediates ADCC and ADCP, with minimal CDC or apoptosis [61, 62], whereas daratumumab is the most effective CDC inducer [27]. The relative contribution of direct apoptosis induction may also vary, with isatuximab showing the strongest pro-apoptotic activity [63]. Importantly, the lysis of target cell depends on the levels of CD38 expression. Beyond that, CD38 expression levels can determine the molecular mechanisms primarily underlying induction of cell death [64].

In transplant settings, daratumumab and isatuximab, as shown in Figure 1, have demonstrated effects on various components of the B cell-driven alloimmune response, depleting plasmablasts, plasma cells, transitional B cells, and memory B cell subsets [44, 58]. These effects likely explain the observed modest reductions in alloantibody levels, consistent with similar findings in daratumumab case studies and series [42–50]. In contrast, felzartamab’s impact on B cell immunity is less defined. A phase 2 trial in late AMR suggested it may not significantly reduce HLA antibody levels [20], but detailed phenotypic and functional studies are awaited.

CD38 monoclonal antibodies rapidly reduce peripheral blood NK cell counts [65], potentially via complement-mediated lysis, NK cell activation leading to exhaustion, and/or FcγRIIIA-mediated fratricide [66]. For isatuximab, transcriptome analyses of NK cells cocultured with myeloma cells revealed deregulated expression of 70 genes linked to chemotaxis, cytolysis, and defense response, reflecting activation via Fc binding and CD38 transmembrane signaling. Labeled NK cell experiments suggested activation followed by exhaustion, rather than fratricide, partially drives NK cell depletion post-isatuximab. Additional studies suggested CD38/SLAMF7-mediated phagocytosis by M2-like macrophages [64]. Whether similar mechanisms apply to felzartamab remains unclear.

CD38 antibodies bind distinct epitopes, leading to variations in their capacity to inhibit CD38’s enzymatic function [27, 31]. This is relevant, as inhibition of the CD38 ectoenzymatic domain has recently been linked to improved T cell metabolic fitness and enhanced T cell cytokine production [67]. The absence of enzymatic inhibition by felzartamab may offer a therapeutic advantage in transplantation by potentially lowering the risk of T cell-mediated rejection (TCMR), a concern linked to other CD38 antibodies like daratumumab [42, 68]. Moreover, CD38 antibodies, including daratumumab, may deplete regulatory B and/or T cells, potentially driving T cell expansion [69]. However, the immunologic consequences of these effects in transplantation remain uncertain. A reported case of early severe TCMR in a kidney transplant recipient treated with daratumumab for myeloma before transplantation underscores this concern [68]. However, in the felzartamab trial transcriptome analyses did not reveal exacerbated TCMR under treatment [20, 70]. Nonetheless, one of the 24-week follow-up biopsies revealed tubulo-interstitial infiltrates classified as Banff IA TCMR after 6 months of treatment. This finding mirrors discrepancies seen with daratumumab, where T cell infiltration occurred despite a negative molecular TCMR score on the Molecular Microscope platform [44].

It remains unclear how differences between CD38 antibodies affect their pharmacodynamic utility, efficacy, and safety in AMR, including risks of infection, malignancy, or TCMR. It is also uncertain whether these differences influence the primary mechanism of action, such as NK cell versus plasma cell depletion.

### Rationale Behind Targeting CD38 in AMR?

The rationale for using CD38 antibodies in AMR lies in the strong expression of CD38 on plasma cells, key producers of alloantibodies. Depleting plasma cells with CD38 antibodies may reduce DSA levels, mitigating rejection. A nonhuman primate study by Kwun et al. [42] showed that rhesus macaques sensitized through sequential skin grafts and treated with daratumumab (combined with plerixafor/anti-CXCR4) had significantly reduced DSA levels and prolonged renal graft survival. Clinically, daratumumab reduced HLA antibodies and improved AMR outcomes in a combined heart/kidney transplant recipient and a highly sensitized heart transplant candidate, facilitating heart graft access [42].

A second rationale for targeting CD38 in AMR, supported by Doberer et al. [44] involves the effect of CD38 monoclonal antibodies on NK cells. This was further confirmed in a phase 2 trial of felzartamab for late AMR, where no meaningful reduction in DSA levels was observed [20]. The effect on NK cell counts is significant, as NK cells are involved in AMR, with studies showing their prevalence in capillaries and association with AMR-related transcripts [71, 72]. Functional polymorphisms in NK cell receptors, such as FcγRIIIA, have also been linked to microvascular inflammation (MVI) in the presence of DSA [73, 74]. NK cell abundance has been identified as a strong predictor of graft outcomes [75]. With rodent studies showing that NK cell depletion can reduce DSA-triggered graft injury [76].

### First Clinical Results of CD38 Targeting in AMR

In recent years, two CD38 monoclonal antibodies, felzartamab and daratumumab, have been used off-label or, in the case of felzartamab, systematically evaluated in AMR through a clinical trial. The results obtained provide an initial look at the potential of these treatments for this complex condition. Table 1 summarizes anecdotal cases, case series, and both completed and ongoing trials. The promising findings, including those from a recently published phase 2 trial in late-stage AMR, will be discussed below.

**TABLE 1:** CD38 targeting in AMR after organ transplantation.

Case reports and case series						
First author, year (ref)	Identifier^a^	CD38 antibody (schedule)	Design	Patients (organ)	AMR phenotype	Key results
Kwun [42]	-	Daratumumab (8 weekly infusions; second 4-month course) plus eculizumab	Case report	1 (heart/kidney)	Rerfractory late AMR plus TCMR (PC-predominant infiltration) Preformed/*de novo* HLA-DSA	Reduction of AMR activity and DSA MFI Depletion of circulating PC, decrease in PC infiltrate Reversal of graft dysfunction Recurrence of rejection after a first treatment course; response to a second course
Spica [43]	-	Daratumumab (6 weekly infusions)	Case report	1 (kidney)	Refractory early AMR ABO-Ab+	Reversal of AMR and ABO Ab reduction Reversal of graft dysfunction
Doberer [44]	-	Daratumumab (IV, 9 months)	Case report	1 (kidney)	Late chronic active AMR HLA-DSA+	Reversal of (histologic/molecular) AMR & DSA reduction Depletion of circulating/intragraft NK cells and bone marrow PC Stabilization of renal function
Süsal [45]	-	Daratumumab (SC, four doses) plus immunoadsorption	Case report	1 (kidney)	Early AMR ABO-Ab+/HLA-DSA+	Reversal of AMR morphology and DSA/anti-A blood group antibody reduction Reversal of graft dysfunction
Zhu [46]	-	Daratumumab (IV; 2–3 months weekly plus PP/IVIG; followed by maintenance with daratumumab alone)	Case series	2 (kidney)	Refractory late/chronic AMR	Resolution of AMR activity (follow-up biopsy in one patient) & DSA reduction Stabilization of renal function Development of TCMR in one patient
De Nattes [48]	-	Daratumumab (IV, 1-weekly/7 weeks); following 1 week immunoadsorption	Case report	1 (kidney)	Early AMR after desensitization HLA-DSA+	Reversal of (histologic/molecular) AMR activity & DSA reduction Stabilization of renal function
Lemal [47]	-	Daratumumab (IV, single dose) Plus PP ± IVIG	Case series	3 (kidney)	Active AMR HLA-DSA+	Resolution of AMR activity and DSA reduction Reversal of graft dysfunction
Vicklicky [50]	-	Daratumumab (SC; 11 injections over 6 months)	Case report	1 (kidney)	Early AMR (subclinical) after desensitization HLA-DSA+	Reversal of (histologic/molecular) AMR & DSA reduction Decrease in dd-cfDNA
Osmanodja [49]	-	Daratumumab (IV; 6–9 months	Case series	2 (kidney)	(Refractory) chronic active AMR HLA-DSA+	Histologic resolution of AMR & DSA reduction Decrease in dd-cfDNA Depletion of circulating NK cells
Guo [77]	-	Daratumumab (IV; 6–19 months), followed by tocilizumab	Case series	7 (kidney)	Late AMR (mixed rejection: n = 5) HLA-DSA+	Stabilization of renal function; reduction in i-IFTA, partial remission of MVI in 4/6 patients at 24–48 months
Systematic trials						
Mayer [20]	NCT05021484	Felzartamab (IV; 0–20 weeks)	Phase 2 trial, randomized, placebo-controlled	22 (kidney)	Late AMR HLA-DSA+	Primary outcome (safety and tolerability): Acceptable safety profile; felzartamab: 8/11 patients with infusion-related reactions Secondary endpoints: Reduction of morphologic/molecular AMR activity (resolution of histologic AMR activity in 9/11 versus 2/11 subjects); NK cell depletion/no effect on DSA levels; dd-cfDNA reduction. (Partial) recurrence of AMR after cessation of a 6-month treatment course
Ongoing trials registered at ClinicalTrials.gov						
-	NCT05913596	Daratumumab (IV; 0–22 weeks)	Single-arm	15	Chronic active AMR HLA-DSA+	Recruiting (Primary outcome measure: percent change in DSA levels)
-	NCT06685757	Felzartamab (IV; up to 12 months)	Phase 3 part 1: 6 months placebo-controlled; part 2: open label)	120	Late AMR HLA-DSA+	Recruiting (Primary outcome measure: percentage of participants who achieve biopsy-proven histologic resolution)

Abbreviations: ABO-Ab, ABO, blood group antibody; AMR, antibody-mediated rejection; dd-cfDNA, donor-derived cell-free DNA; DSA, donor-specific antibody; IV, intravenous; IVIG, intravenous immunoglobulin; MFI, mean fluorescence intensity; NK, natural killer cell; PC, plasma cells; PP, plasmapheresis; SC, subcutaneous; TCMR, T cell-mediated rejection.

^a^ ClinicalTrials.gov identifiers.

### Felzartamab in Late AMR

**FIGURE 2:**
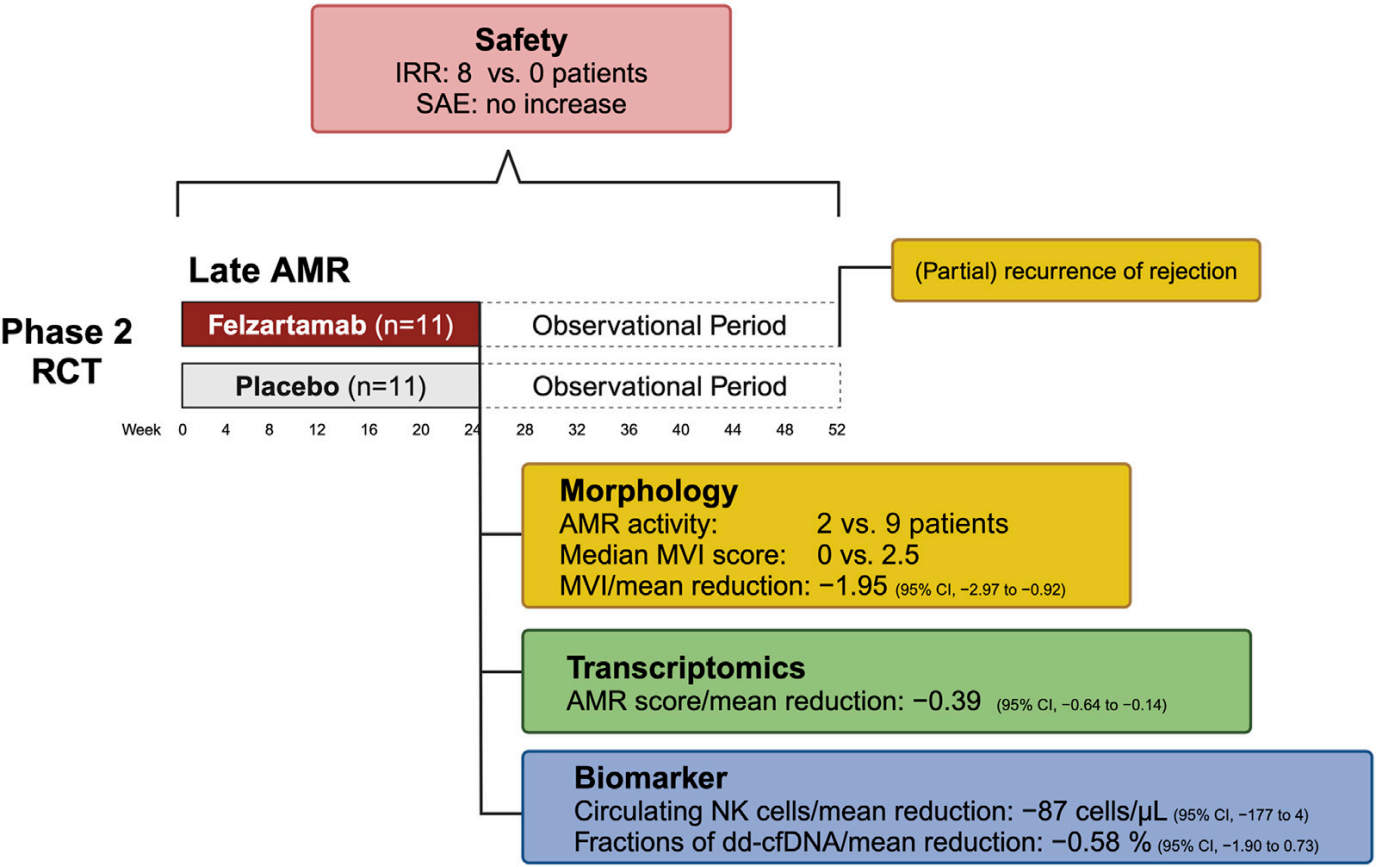
Key results from the phase 2 trial of felzartamab in late antibody-mediated rejection. Abbreviations: AMR, antibody-mediated rejection; CI, confidence interval; dd-cfDNA, donor-derived cell-free DNA; IRR, infusion-related reactions; MVI, microvascular inflammation; NK cell, natural killer cell; RCT, randomized controlled trial.

A recent randomized, placebo-controlled phase II pilot trial evaluated a six-month treatment course of the CD38 antibody felzartamab versus placebo in late DSA positive AMR ≥180 days after kidney transplantation, demonstrating significant reductions in AMR activity as observed in 24-week follow-up biopsies [20]. Key secondary outcomes underscored felzartamab’s effectiveness, with 82% of patients achieving resolution of AMR activity by week 24, compared to 20% in the placebo group, alongside notable reductions in MVI scores [20]. Major results of the felzartamab trial are shown in Figure 2.

Transcriptomic studies showed that felzartamab consistently reduced molecular AMR activity scores by selectively suppressing interferon gamma-inducible and natural killer cell transcripts, with minimal effects on AMR-induced endothelial transcripts [70]. However, while therapy reduced AMR activity in all subjects who had pretreatment activity, the suppression was often incomplete in those with very high activity. While MVI recurred in only a subset of patients at week 52, molecular recurrence was nearly universal after treatment discontinuation. Interestingly, molecular analyses indicated that felzartamab provided sustained parenchymal benefits, slowing the progression of molecular injury even after the treatment period [70]. Importantly, resolution of AMR activity was linked to rapid and substantial reductions in donor-derived cell-free DNA (dd-cfDNA) —a marker of ongoing allograft injury—though levels approached baseline after treatment cessation [20].

While the results suggest that targeting CD38 may have the potential to slow the progression to kidney failure, the trial was not powered to assess the impact of felzartamab on long-term graft outcomes [78]. However, preliminary data, including an apparent stabilization of eGFR slope, hinted at potential clinical benefit [20]. An analysis of the iBox prognostication system designed to predict death-censored allograft survival [79] revealed that the probability of graft survival decreased by 7.6% per year in the placebo arm, and increased by 6.4% in the felzartamab arm [80]. Remarkably, in an analysis evaluating the two trial periods (6 months treatment; 6 months observation) this treatment effect was observed only during the exposure period [80]. However, it should be noted that the actual value of the iBOX scoring system, or similar models, in predicting survival for such a new intervention, needs to be validated using real survival data.

The treatment with felzartamab was associated with an 80% reduction in CD16^bright^ NK cells and 20%–30% decreases in immunoglobulin levels, though no significant changes were observed in immunodominant DSA levels [20]. While causality has yet to be confirmed, these findings suggest that depleting FcR-expressing NK cells may disrupt the pathogenic effects of DSA in the allograft microvasculature, potentially mitigating DSA-mediated injury. Despite the trial’s limited sample size and short treatment duration, the results are promising, especially when compared to previous trials like the IMAGINE study, which was prematurely halted due to lack of clinical efficacy (ClinicalTrials.gov identifier, NCT03744910). From a commercial perspective, the CD38 antibody landscape is highly competitive, with several therapies targeting plasma cells or immunoglobulin kinetics. However, felzartamab’s distinct mechanism—focusing on CD38^+^ FcR-expressing NK cells and to a lesser extent CD38^+^ plasma cells—could provide a unique clinical differentiation.

Felzartamab demonstrated an acceptable safety profile [20]. Adverse events were more frequent in the felzartamab group (119 events) compared to the placebo group (81 events), though serious adverse events were less common in the felzartamab group (9% vs 36%). Mild to moderate infusion-related reactions (IRR) occurred in 73% of felzartamab-treated patients, despite premedication. Infections were more common in the felzartamab group (91%) than in the placebo group (64%), with nasopharyngitis being the most frequent. However, no serious infection-related adverse events were observed. Additionally, no safety signals were seen regarding COVID-19, with only mild or moderate infections in the felzartamab arm, while the placebo arm had two COVID-19-related serious adverse events [20]. Interestingly, a previous report in membranous glomerulopathy showed robust immune responses in felzartamab-treated patients following SARS-CoV-2 vaccination [81].

Anti-drug antibodies (ADA) were not observed [20, 82]. It is worth noting that felzartamab has primarily been studied in European populations, and potential racial differences in ADA development warrant further exploration [82].

### Felzartamab - Regulatory Designation and Future Prospects

Felzartamab received Orphan Drug Designation for AMR treatment from the U.S. Food and Drug Administration (FDA) in March 2024 and the European Commission in December 2024. The FDA also granted Breakthrough Therapy Designation in October 2024. In a phase 2 trial, felzartamab showed an acceptable safety profile and promising efficacy, though AMR recurrence occurred after therapy discontinuation. Ongoing studies are exploring longer or individualized treatment regimens (based on dd-cfDNA monitoring), with a phase 2 extension trial (NCT05021484) assessing repeated felzartamab courses in patients with recurrent or persisting AMR. Further confirmation of its safety and efficacy in larger patient cohorts is needed. Additionally, a phase 3 trial (TRANSCEND) in late AMR has been launched in the US and is expected to start in Europe (NCT06685757).

### Daratumumab in AMR–Anecdotal Cases, Case Series, and Ongoing Trials

A series of case reports and small case series highlight the efficacy of CD38 targeting in managing AMR after kidney transplantation in diverse scenarios, including early, late, and treatment-refractory rejection (Table 1). Kwun et al. [42] reported a case of DSA-positive AMR following kidney and heart transplantation, showing reductions in both AMR activity and DSA-MFI. Spica et al. [43] reported successful resolution of early AMR after ABO-incompatible transplantation, with a notable decrease in anti-A blood group antibodies. In a case of chronic active AMR, Doberer et al. [44] demonstrated persistent resolution of rejection activity, accompanied by plasma cell and NK cell depletion as well as reduced DSA levels and altered DSA production by antibody secreting cells isolated from bone marrow aspirates. Süsal et al. [45] described early, DSA-positive AMR occurring 5 days post-ABO and HLA-incompatible transplantation, again with reductions in anti-A blood group titers and DSA levels. Zhu et al. [46] presented the course of daratumumab plus PP/IVIG in two patients with refractory chronic active AMR showing reduced DSA-MFI, while Lemal et al. [47] reported AMR resolution and DSA-MFI reductions in three AMR cases. In another case, de Nattes et al. [48] reported on a sensitized patient who after successful transplantation under desensitization showed AMR in a 3-month biopsy. Rejection was successfully reversed using daratumumab, as also supported by molecular analysis [48]. Viklicky et al. [50] described histologic and molecular AMR resolution with decreased DSA-MFI, and Osmanodja et al. [49] reported reductions in AMR activity, NK cell depletion, and moderate DSA-MFI reductions in two cases of refractory DSA-positive AMR. In the latter two reports, also a substantial decrease in dd-cfDNA levels was documented [49, 50]. These findings collectively underscore the potential of CD38 targeting in resolving histologic (and as shown in two cases also molecular) AMR activity and mitigating immune-mediated injury. While not proven in a rigorous study, daratumumab may affect alloantibody levels to a certain degree. Given the proposed deregulation of T cell immunity it is notable that clinically relevant TCMR was reported in one of the reported cases [46], while in another case subclinical CD3^+^ T cell infiltrates were noted but not associated with molecular TCMR-related transcript sets [44]. A prospective open-label, single-arm trial of daratumumab in DSA-positive chronic active AMR, with DSA MFI reduction as the primary outcome, is underway in China (ClinicalTrials.gov identifier: NCT05913596) (Table 1). Finally, a recent case series including seven patients with late or chronic active AMR suggests the potential of sequential therapy with CD38 mAb followed by tocilizumab to enhance DSA reduction [77].

While the aforementioned anecdotal reports and case series provide interesting initial insights, we emphasize that systematic trials will ultimately be necessary to establish the efficacy of daratumumab in AMR, whether as monotherapy or in combination with other therapies. Such studies may clarify the ability of daratumumab to decrease DSA, further assess its safety—particularly its potential role in triggering TCMR (which was not a safety issue with felzartamab in the phase 2 trial)— and determine the clinical significance of differences in the mechanisms of action among CD38 antibodies. Variations in efficacy or safety could stem from differing effects on immune cell subpopulations due to variable complement-fixing ability or ectoenzyme interference in relation to ADCC or ADCP induction.

## CD38 Antibodies for DSA- and C4d-Negative MVI?

In the Banff 2022 scheme, a distinct MVI subcategory, that is, MVI, C4d-negative and DSA-negative, was defined [12]. Gene expression patterns associated with this phenotype were found to be similar to those observed for AMR [83]. However, in addition to potential antibody-triggered mechanisms, including non-HLA specificities, mechanisms independent of DSA—such as missing-self NK cell activation or alloantigen-dependent NK or monocyte activation—have been proposed [10]. Given the potential key role of NK cells in these cases, one might speculate that CD38-targeting therapy could be beneficial, where other treatments may be less effective. As an example, a recent cohort study suggested that in DSA-MVI, tocilizumab, in contrast to DSA + rejection, failed to modify the course of eGFR, possibly due to persistent NK cell activity [84]. Future studies may be of interest to explore whether CD38 targeting could be effective in such cases.

## CD38 Antibodies for the Prevention of AMR in Pre-Immunized Patients?

CD38 antibodies may aid in the transplantation of highly immunized patients in two ways. First, depletion of HLA antibody-producing plasma cells can be expected to gradually decrease the levels of preformed deleterious alloantibodies and increase the chance to receive a suitable organ. On the other hand, pre-emptive depletion of the effector cell population (CD38^+^ NK cells) may also be clinically beneficial to limit ADCC and ADCP in the early posttransplant phase. The latter concept is supported by a report by Schrezenmeier et al. [85] who documented successful prevention of rejection with daratumumab (single dose shortly before transplantation, thereafter continued treatment), combined with imlifidase, intravenous immunoglobulin and rituximab-based desensitization to allow T- and B-cell cytotoxic crossmatch-positive and ABO-incompatible living donor transplantation in a 35-year-old female patient systemic lupus erythematosus (SLE) and antiphospholipid syndrome. The patient exhibited an extreme level of HLA sensitization and was running out of vessels. Two follow-up biopsies showed no features of AMR, perhaps not only the effect of transient antibody depletion, but also a result of a continuous depletion of NK effector cells [85].

Several studies have explored the use of CD38 monoclonals, particularly daratumumab and isatuximab, for recipient desensitization to reduce preformed HLA antibodies. Case reports and small case series [42, 47, 86] have supported ongoing trials addressing this issue. Two trials, one using isatuximab and the other daratumumab, have been published. In an open-label phase 1/2 study, Vincenti et al. [57] investigated the safety, pharmacokinetics, and preliminary efficacy of isatuximab in patients awaiting kidney transplantation. The study included 23 patients who received isatuximab 10 mg/kg weekly for 4 weeks then every 2 weeks for 8 weeks. Treatment was well tolerated and resulted in decreases in CD38^+^ plasmablasts, plasma cells, and NK cells and significant reductions in HLA-specific IgG-producing memory B cells. Treatment decreased HLA antibodies to a certain extent, an effect that was maintained for 26 weeks after the last dose. Overall, calculated panel reactive antibody (cPRA) values were only minimally affected, but six patients received transplant offers, of which four were accepted. In a prospective 2-phase monocenter open-label trial (DARDAR study), Pilon et al. [87] investigated the safety and efficacy of daratumumab in kidney transplant candidates >95% cPRA. In the first (safety) phase (9 patients), they used 4-weekly escalating doses of daratumumab. Phase 2 tested desensitization with 8 weekly infusions (14 patients). Treatment-emergent adverse events were mostly infusion-related, with no serious adverse events reported. The study showed significant, though transient and incomplete reductions in cPRA levels and the number and MFI of HLA antibodies at 6 months. The modest effect on HLA antibodies was temporary, with levels returning to baseline after 12 months. The authors highlighted this as a limitation for the clinical use of daratumumab for desensitization.

Torija et al [58] further analyzed 26 highly sensitized patients from the two CD38 antibody desensitization trials [57, 87], confirming the significant depletion of plasmablasts, long-lived plasma cells, and other B cell subsets, including B cell precursors and class-switched memory B cells. They identified key phenotypes, particularly CD38-negative class-switched memory B cells, differentiating successful serologic responders from low- or non-responders [58].

Strategies to enhance HLA antibody reduction by targeting CD38, such as combining daratumumab with belatacept, are under investigation (ClinicalTrials.gov identifiers: NCT04827979; NCT05145296).

## Conclusion

Antibody-mediated rejection (AMR) remains a significant challenge in kidney transplantation, as current treatments show low levels of evidence and inconsistent outcomes. CD38-targeted therapies, including daratumumab and felzartamab (in a recent phase 2 trial), have shown promise in AMR management. Felzartamab demonstrated an acceptable safety profile and resolution of rejection in many recipients, though the effects were transient, with partial recurrence of rejection. Modulation of dd-cfDNA and gene signatures indicating an injury/repair response in the felzartamab trial suggest potential long-term graft benefits. However, the value of dd-cfDNA as a non-invasive monitoring tool for detecting treatment responses and AMR recurrence still needs to be proven in larger trials. The mechanisms underlying CD38-targeted therapy efficacy remain unclear, and the dual-action model targeting plasma cells and NK cells may oversimplify its therapeutic mode of action. Ongoing studies, including a phase 3 trial, are crucial to confirm the impact on AMR and assess the long-term benefits of CD38-targeting therapies, including their broader potential in kidney transplantation and recipient desensitization.
